# Protocol to establish mouse esophageal epithelial whole mounts for lineage tracing or immunofluorescent analysis

**DOI:** 10.1016/j.xpro.2024.103340

**Published:** 2024-09-25

**Authors:** David Grommisch, Wei Yang, Maria Genander

**Affiliations:** 1 Department of Cell and Molecular Biology, Karolinska Institutet, 17165 Solna, Sweden

**Keywords:** Antibody, Stem Cells, Cell Differentiation

## Abstract

Herein, we provide a protocol describing the preparation and labeling of cells in mouse esophageal epithelial whole mounts. We include details of tamoxifen administration used to genetically label and trace the fate of basal cells. We demonstrate how EdU incorporation alone or in combination with additional markers can be used to assess *in vivo* cell cycle progression or stratification dynamics. Finally, we highlight how optimization of cell labeling is required for reproducible analysis.

For complete details on the use and execution of this protocol, please refer to Grommisch et al.^1^

## Highlights

• Protocol for establishing mouse esophageal epithelial whole mounts
• Guide to genetic recombination and tracing using topical tamoxifen administration
• Detailed steps describing how to combine EdU and antibody labeling

## Abstract (editor highlights)

Publisher’s note: Undertaking any experimental protocol requires adherence to local institutional guidelines for laboratory safety and ethics.

## Graphical abstract

**Figure:**
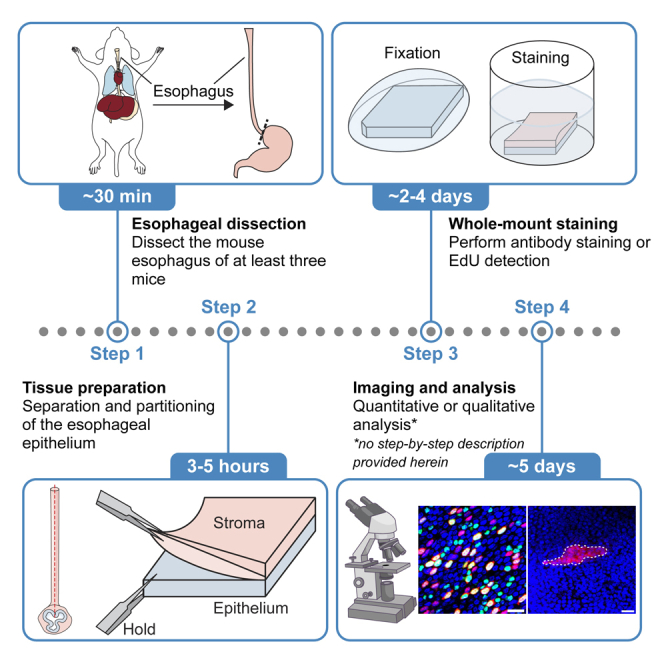

## Before you begin

This protocol delineates the specific steps required to prepare mouse esophageal whole mounts for immunofluorescent staining or visualization of reporter-positive clones in lineage tracing experiments. We present the protocol which was used to compare unbiased and targeted quantitative lineage tracing data in Grommisch et al.,^1^ building on previous work establishing esophageal epithelial whole mounts.^2^ In addition to analyzing long-term lineage tracing experiments,^2^^,^^3^^,^^4^ we show how whole mounts represent an excellent model to quantitatively describe epithelial cell cycle states and stratification rates. Furthermore, we showcase how employing a combination of basal (CD49f, CYCLINA2 and EdU) as well as suprabasal (KLF4) markers provide information of epithelial cell states.

### Institutional permissions

All animal experiments were approved by the appropriate ethical review board (Stockholms Norra Djurförsöksetiska Nämnd, ethical permits 14051–2019 and 735–2021). Relevant institutional permission must be acquired prior to commencing the following protocol. When possible, adhere to the 3R principles of replacement, reduction and refinement when conducting animal research.

### Tamoxifen solution and topical application

**Timing: 15 min preparation; 30 min topical application**

Here, we describe the steps for preparing and applying tamoxifen.

Tamoxifen diluted in ethanol is applied topically on the back skin of mice.^1^ Shaving of the back skin is not required for efficient labeling of the esophageal epithelium. Single-housed mice are equally well recombined as mice housed together, indicating that Tamoxifen is taken up through the skin and not primarily by licking at the applied skin area. Other studies have reported similar lineage tracing data using Tamoxifen diluted in corn oil and injected intraperitoneally.^2^^,^^3^ In our hands, topical application however works equally well and is judged to be less stressful for the animals.

1. Weigh Tamoxifen powder.
  a. Begin by weighing an appropriate amount of Tamoxifen for your experiment on a microscale. Tamoxifen powder is stable for up to two years when stored at 4°C in the dark.
2. Reconstitute in absolute ethanol.
  a. Reconstitute 100 mg Tamoxifen powder in 10 mL absolute ethanol to generate a 1% Tamoxifen in ethanol solution (W/V). If required, generate a lower or higher concentration by adjusting the amount of Tamoxifen powder.
  b. Vortex the solution for 2–5 min checking frequently if the Tamoxifen is fully dissolved.
  c. Wrap the dissolved Tamoxifen solution in aluminum foil to protect the solution from light.
  d. Label the tube with the health hazard symbol for CARCINOGENS. Tamoxifen solution can be stored for up to a month at 4°C. Long term storage (<1 year) can be done at −20°C.

**CRITICAL:** Tamoxifen is cancerogenic. Always work in a fume hood with the necessary protection (googles, nitrile gloves, lab coat, mask) and avoid skin contact or inhaling the powder. Make sure no precipitates or undissolved Tamoxifen remains to ensure full dissociation of the Tamoxifen and even labeling between mice.

3. Topical back skin administration of Tamoxifen.
  a. Always look for Tamoxifen precipitates before treating the mice. If you observe precipitates, warming up the Tamoxifen solution can help dissociation. If no precipitates are present, pre-warming of Tamoxifen is not required. Hold the mouse at the tail and apply 200 μl of Tamoxifen in ethanol solution topically to the back skin using a 1 mL pipette. It is essential to optimize the concentration of Tamoxifen required for recombination of single basal cells in each CreER^T2^ mouse line. 200 μl of 1% Tamoxifen or 200 μl of 0.02% Tamoxifen is enough to induce sparse recombination in Troy-CreER^T2^ and Sox2-CreER^T2^ mice, respectively.^1^
    **CRITICAL:** Separate mice treated with Tamoxifen from untreated littermates. Grooming and excreted Tamoxifen could recombine mice in the same cage that were not treated.
  b. Wait until the experimental endpoints are reached [∼up to 1 year].

### Prepare the blocking solution

**Timing: 1 h**

Here, we describe how to prepare the blocking solution used for antibody stainings.

4. For 50 mL blocking solution.
  a. Prepare a 10% Triton X-100 solution (V/V) by pipetting 1 mL Triton X-100 to 9 mL DPBS. The solution can be stored for 1 month at room temperature (RT).
  b. Weigh 0.5 mg Bovine serum albumin (BSA) in a 50 mL falcon tube.
  c. Add 20 mL 1X DPBS and 2.5 mL of the 10% Triton X-100 solution, for a final concentration of 0.5%.
  d. Add 2.5 mL of Normal Donkey Serum (NDS).
  e. Put solution on a rocket shaker or carefully shake to prevent formation of foam but allow mixing. Make sure that all precipitates are gone. Once all components are dissolved fill the tube to 50 mL with DPBS. The blocking solution can be stored for up to one month at 4°C.

### Prepare stock solutions for EdU detection

**Timing: 1 h**

Here, we describe how to prepare the stock solutions used to make the final EdU detection solution.

Required stocks and reagents for EdU detection.

100 mM CuSO_4_ (Copper (II) sulfate).

1 M Ascorbic Acid (AA) (100X).

2 mM Alexa Fluor 488 Azide.

5. For a 100 mM CuSO_4_ solution dissolve 15.961 mg CuSO_4_ per 1 mL dH_2_O. Store at 4°C.
6. For a 1 M Ascorbic acid solution dissolve 176.12 mg L-Ascorbic Acid per 1 mL dH_2_O. Aliquot and store at −20°C. Avoid freeze and thaw cycles.
7. For a 2 mM Alexa Fluor azide solution dissolve 1.7 mg of Alexa Fluor 488 Azide in 1 mL Dimethylsulfoxide (DMSO). Aliquot and store at −20°C.

***Note:*** Alexa Fluor 488 Azide is our choice here since we use a red channel endogenous fluorescent reporter (tdTomato). If different channels are required, other fluorophores such as Alexa Fluor 647 Azide can be used.

### Prepare EdU detection solution

**Timing: 15 min**

Here, we describe how to prepare the EdU detection solution used to visualize EdU-positive cells in the epithelial whole mounts.

8. Calculate the required amount of EdU detection solution. For each piece use 300 μl when using a 24-well plate. As an example, an 1 mL EdU detection mix is described below.
  a. Add 859.5 μl DPBS to a 1.5 mL reagent tube.
  b. Add 40 μl of 100 mM CuSO_4_.
  c. Add 0.5 μl of 2 mM Alexa Fluor 488 Azide to the DPBS and flick the tube to mix.
  d. In another reagent tube prepare 10X L-Ascorbic Acid solution and dilute 10 μl of the 100X AA solution in 90 μl of DPBS and vortex until any potential precipitates are completely dissolved.
  e. Add the 10X AA solution to the reagent tube with the DPBS, CuSO_4_, and azide fluorophore and pipette up and down to mix.

**CRITICAL:** Once the AA solution is added the reaction starts. Therefore, do not prepare the EdU detection solution more than 15 min prior to use.

## Key resources table

**Table:** 

REAGENT or RESOURCE	SOURCE	IDENTIFIER
**Antibodies**		

Rabbit anti-recombinant anti-KLF4, clone EPR20753-25, dilution 1:400	Abcam	Cat#ab214666; RRID:AB_2943042
Rat anti-CD49f (Integrin alpha 6) monoclonal antibody, clone eBioGoH3 (GoH3), dilution 1:500	eBioscience	Cat#14-0495-82; RRID:AB_891480
Rabbit anti-CYCLIN A2, clone EPR17351, dilution 1:400	Abcam	Cat#ab181591 RRID: AB_2890136
Donkey anti-rabbit 488, dilution 1:1,000	Jackson ImmunoResearch Laboratories	Cat#711-545-152; RRID:AB_2313584
Donkey anti-rat 488, dilution 1:1,000	Jackson ImmunoResearch Laboratories	Cat#712-545-153; RRID:AB_2340684
Donkey anti-rabbit 647, dilution 1:1,000	Jackson ImmunoResearch Laboratories	Cat#711-605-152; RRID:AB_2492288

**Chemicals, peptides, and recombinant proteins**		

Tamoxifen	Sigma-Aldrich	Cat#T5648-5G
Ethanol	Histolab	Cat#4020309
Triton X-100 solution	Merck	Cat#93443-100ML
DAPI (4′,6-diamidino-2-phenylindole, dihydrochloride)	Thermo Fisher Scientific	Cat#D1306
Bovine serum albumin	Sigma-Aldrich	Cat#A4503 100 g
Normal donkey serum	Jackson ImmunoResearch Laboratories	Cat#017-000-121
EDTA	VWR	Cat#E522-100ML
DPBS	Thermo Fisher Scientific	Cat#14190250
HBSS (no calcium, no magnesium)	Thermo Fisher Scientific	Cat#14175-129
ProLong Gold antifade mountant 10 mL	Thermo Fisher Scientific	Cat#P36930
CuSO_4_	Sigma-Aldrich	Cat#451657-10G
L-ascorbic acid	Sigma-Aldrich	Cat#A4544
Alexa Fluor 647 Azide.	Thermo Fisher Scientific	Cat#A10277
Alexa Fluor 488 Azide	Thermo Fisher Scientific	Cat#A10266
5-Ethynyl-2′-deoxyuridine	Sigma-Aldrich	Cat#900584-50MG
Dimethyl sulfoxide	Sigma-Aldrich	Cat#276855-100ML
Tween 20, 100% non-ionic detergent	Bio-Rad	Cat#1706531
Formaldehyde 37% (with 10%–15% methanol)	Sigma-Aldrich	Cat#252549-1L

**Critical commercial assays**		

Click-iT EdU Cell Proliferation Kit for Imaging, Alexa Fluor 488 dye	Thermo Fisher Scientific	Cat#C10337

**Experimental models: Organisms/strains**		

Mouse: TroyCreERT2;tdTomato, males and females over 80 days of age	Hans Clevers, Hubrecht Institute	Stange et al.^5^
Mouse: B6;129S-Sox2tm1(cre/ERT2)Hoch/J, males and females over 80 days of age	Jackson Laboratory	JAX: 017593

**Software and algorithms**		

Fiji	2.9.0/1.53t	https://imagej.net/software/fiji/

**Other**		

Petri dish, 92 × 16 mm	Sarstedt	Cat#82.1472.001
Spring scissors angled to side ball tip 8 mm cutting edge	Fine Science Tools	Cat#15033-09
Sterile stainless steel scalpels, blade no. #15	VWR	Cat#76044-672
SuperFrost slides	VWR	Cat#631-9483
Menzel Deckgläser/coverslips	Fisher Scientific	Cat#Q10143263NR15
SafeSeal reaction tube, 1.5 mL	Sarstedt	Cat#72.706
Cell culture plate, 12 well, surface: standard, flat base	Sarstedt	Cat#83.3921.005
Cell culture plate, 24 well, surface: standard, flat base	Sarstedt	Cat#83.3922.005
Cell culture plate, 48 well, surface: standard, flat base	Sarstedt	Cat#83.3923.005
Screw cap tube, 15 mL	Sarstedt	Cat#62.554.016
Screw cap tube, 50 mL	Sarstedt	Cat#62.547.254
Dumont #5 forceps, biology tip	Fine Science Tools	Cat#11252-20
Rocket shaker	–	–
Incubator	–	–
Stereo microscope	–	–

## Materials and equipment

**Table:** Blocking buffer mix

Reagent	Final concentration	Amount for 50 mL
Bovine serum albumin	1% (W/V)	50 mg
Triton X-100 (10%)	0.5% (V/V)	2.5 mL
Normal Donkey Serum	2.5% (V/V)	2.5 mL
DPBS	N/A	45 mL
Total	N/A	50 mL

At 4°C the blocking buffer can be stored for up to 1 month.

**Table:** Stock solutions for homemade EdU detection

Reagent	Final concentration	Amount for 1 mL
Copper (II) sulfate (CuSO_4_)	100 mM	15.961 mg (dH_2_O)
L-Ascorbic Acid (AA)	1 M (100X)	176.12 mg (dH_2_O)
Alexa Fluor 488 Azide	2 mM	1.7 mg (DMSO)

At 4°C the blocking buffer can be stored for up to 1 month.

**Table:** EdU detection reaction mix

Reagent	Final concentration	Amount
DPBS	N/A	859.5 μl
CuSO_4_	4 mM	40 μl
Azide flurophore	1 μM	0.5 μl
L-Ascorbic acid (1X)	10 mM	100 μl (90 μl dH_2_O + 10 μL 100X L-Ascorbic acid)
Total	N/A	1 mL

**CRITICAL:** Prepare detection mix in the given order not more than 15 min before use. Vortex the 100X L-Ascorbic Acid solution before use since the L-Ascorbic acid might precipitate during thawing due to the low temperatures. Ensure that the L-Ascorbic acid is completely dissolved before you proceed. Pre-dilute to 10X before mixing. Once the L-Ascorbic acid is added the Click-iT reaction will start.

Instead of the homemade EdU detection reaction you can use the commercial Click-iT EdU Cell Proliferation Kit for Imaging, Alexa Fluor 488 dye (Thermo Fisher #10348924) and follow the instructions found here (Online protocol). Since large volumes of EdU detection reagent are needed the homemade solution is a cost-efficient solution compared to commercially available kits.

## Step-by-step method details

### Dissection and separation of mouse esophagus

**Timing: 5 h**

In this step we describe how the esophagus is dissected and how the epithelium is separated from the stroma,^6^ We also describe how the tissue is prepared for staining and imaging. This step can be applied to mice of all postnatal ages. Here we use mice older than 70 days, since the postnatal growth of the esophagus is then complete.^7^

**Figure 1:**
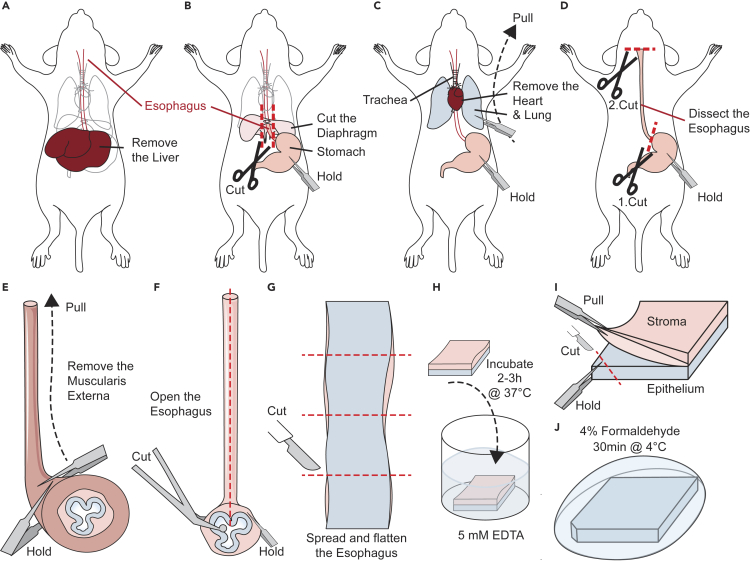
Schematic representation of dissection and isolation of the esophageal epithelium (A) Opened mouse carcass displaying the position of the liver that should be removed. (B) Visualization of the stomach and diaphragm. The diaphragm is removed by slightly pulling the stomach downwards and cutting sideways along the esophagus. (C) Visualization of the lungs and trachea after removing the diaphragm. Maintain a slight pull of the stomach and use a second pair of forceps to grab and remove the lungs and trachea in an upwards movement. (D) Grab the distal end of the esophagus and cut the esophagus close to the stomach. Cut at the proximal end of the stomach to release the esophagus. (E) Mechanical removal of the muscularis externa using two pair of forceps. (F) Use ball-tip scissors to open the mouse esophagus (epithelium and submucosa) longitudinally. (G) Cut the flat mouse esophagus in equally sized fragments. (H) Submerge each esophageal fragment in 5 mM EDTA. (I) Separate the esophageal epithelium from the submucosa. (J) Fix the epithelial whole mount in 4% formaldehyde. Note that the incision in the lower left corner made by the forceps when separating the epithelium from submucosa can aid in tissue orientation in later steps. A video demonstration of the dissection is previously published.^6^

**Figure 2:**
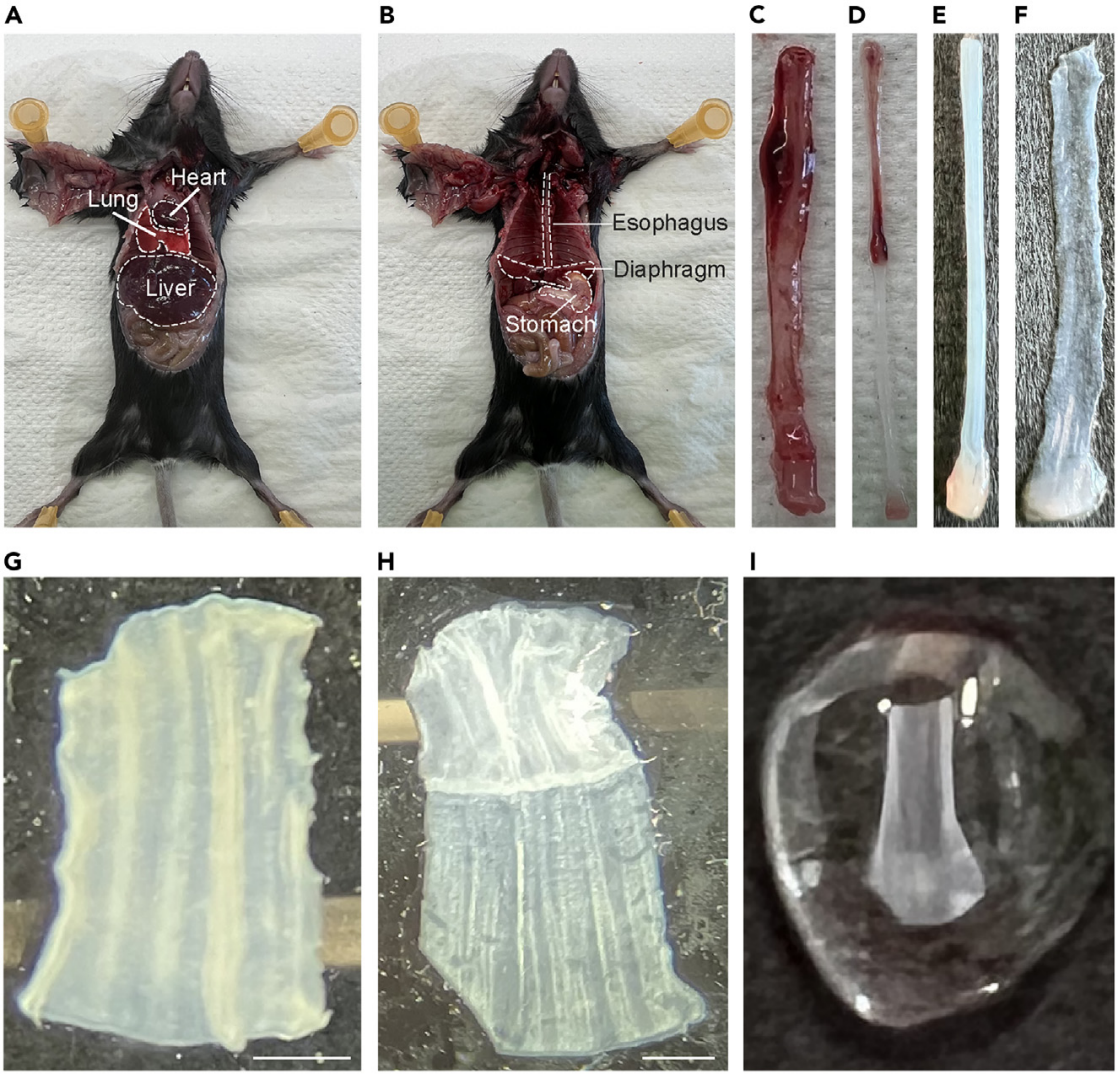
Images from dissection and isolation of the esophageal epithelium (A) Opened mouse carcass displaying the position of the liver, lung, and heart, which should all be removed. (B) Opened mouse carcass displaying the position of the diaphragm, which should be removed, as well as the stomach and esophagus. (C) Dissected esophagus still surrounded by the muscularis externa. (D) Image of dissected esophagus after mechanically stripping the muscularis externa. Muscularis externa (upper dark part) is still attached to the rest of the esophageal tube (lower transparent part). (E) Higher resolution image of the esophageal epithelium surrounded by stroma. (F) Picture displaying a longitudinally opened and flattened esophagus (epithelium and stroma). (G) High resolution image of one section of the esophagus comprised of the stromal and epithelial layers before treatment with 5 mM EDTA. Scale bar = 1 mm. (H) Esophageal epithelial fragment (lower transparent part) after removal of esophageal stroma (upper white part). Note the incision in the lower left corner. Scale bar = 1 mm. (I) Epithelial whole-mount fragment submerged in a 4% formaldehyde drop.

***Note:*** In all further steps ensure that the esophagus remains moist and is always covered in DPBS.

1. Sacrifice mice using CO_2_ (or a method in line with local ethical regulations).
2. Dissect the esophagus (Figures 1 and 2).
  a. Open the abdomen of the mouse (Figures 1A and 2A).
  b. Locate the liver (Figures 1A and 2A).
    i. Remove the liver.
  c. Locate the stomach (Figures 1B and 2B).
    i. Gently pull the stomach using surgical tweezers to reveal the connection to the esophagus.
    ii. Use surgical scissors to cut the diaphragm alongside the esophagus.
    iii. While gently pulling the stomach use a second tweezer to pull away the lung and trachea in an upward movement.
  d. Hold the end of the esophagus and cut closely to the stomach (Figures 1C and 1D).
  e. While holding onto the distal end, cut the proximal end of the esophagus before it becomes the pharynx (Figure 1D).
  f. Submerge the esophagus in cold DPBS and place it on ice.

3. Mechanically remove the external muscle layer under a stereoscope.
  a. Hold the distal end of the esophagus with a surgical tweezer and use the second surgical tweezer to gently grab the external muscle layer. In an upward movement gently pull away the muscle layer (Figures 1E, 2C, and 2D).
  b. There is usually some muscle left around the distal esophagus where the tweezer was placed in (a).
  c. To remove the leftover muscle at the distal end of the esophagus use a surgical tweezer to gently squeeze the middle tissue of the esophagus and use the second fine-tipped tweezer to remove the leftover muscle layer at the distal esophagus.
4. Use the ball-tip spring scissor to open the esophagus longitudinally. Insert the ball-tip at the distal end of the esophagus and carefully cut along the esophageal axis (Figures 1F, 2E, and 2F).
5. Cut the esophagus in approximately 0.4–0.5 cm pieces (Figure 1G).

***Note:*** It is also possible to leave the entire esophageal tube intact, although some of the subsequent steps will become more difficult and the immunofluorescent labeling may become variable.

6. Submerge each piece of the esophagus in one well of a 24-well plate filled with 300 μl 5 mM EDTA in DPBS (Figure 1H).
7. Place the 24-well plate in a 37°C incubator equipped with a rocker shaker for 2–3 h.
8. Remove the esophageal segments from the solution and place in a clean 10 cm Petri dish.
  a. Cover the tissue piece in a small amount of DPBS to prevent it from drying out.
  b. Orient the esophagus so that the apical (originally facing the lumen) epithelial side faces the bottom of the dish.
  c. Hold on to one corner of the esophageal fragments with one surgical tweezer and use the second to carefully pull off the stroma.
  d. Use a scalpel to make a small incision or cut to the epithelium (Note: Ideally cut the piece you held on to) to know the fragments orientation (Figures 1I, 2G, and 2H). Troubleshooting 1 and 2.

***Note:*** This protocol focuses on the esophageal epithelium, but the stroma can be processed in a similar way as described for the esophageal epithelium.

**CRITICAL:** The epithelium and stroma can be identified by optical inspection. While the epithelium is transparent the stroma is slightly opaque and has a “more flexible structure” as previously visualized.^6^

9. Use a Kimwipe to remove excess liquid from the tissue.

***Note:*** This is easier if you slightly tilt the Petri dish

10. Apply 50–80 μl of 4% formaldehyde (with 10%–15% methanol) in 1 x DPBS buffer for 30 min at 4°C. Use a stereoscope to ensure that the epithelial pieces are fully submerged in the fixing solution.

**CRITICAL:** If you observe floating tissue pieces use surgical tweezers to gently submerge the pieces. Inadequate fixation can lead to suboptimal antibody staining.

***Note:*** Fixation can also be performed for 30 min at 20°C–23°C., which is expected to increase fixation efficiency.^2^ Fixation time can also be increased to improve labeling with specific antibodies.

11. Wash the tissue piece twice with 1 x DPBS.

***Note:*** If the epithelial whole mounts are derived from lineage traced esophagi, fluorescent reporter positive (e.g tdTomato, RFP and YFP) clones can be visible without additional antibody staining. Then proceed to step 19 for mounting, but esophagi expressing fluorescent reporters should be kept dark (covered with foil) during procedures to avoid bleaching.

### Immunofluorescent labeling and EdU detection in epithelial whole mounts

**Timing: 2–4 days**

Here we describe how to co-label reporter-positive lineage traced cells with additional markers of interest, enabling characterization of traced cells. It is also possible to label wild type whole mounts with antibodies of interest. Labeling with progenitor cell markers, markers for differentiated cells or EdU can aid in determining progenitor cell behavior and states. The analysis and quantification are normally done manually while looking through the microscope. However, tools such as Imaris or ImageJ can aid in quantification of acquired images.

When applying new primary antibodies, optimizing the fixation incubation time is required. Staining with antibodies targeting transcription factors can commonly be improved using a prolonged incubation time (up to 72 h). All subsequent steps required for successful staining are carried out in either 24-well or 48-well plates. For all incubation steps the plates are kept on a rocker or orbital shaker. To change medium in the wells, use a P200 pipette to carefully remove and add solution. Tilting the plate might help to remove all liquid. Avoid excessive touching of the epithelial fragment with the pipette.

12. Incubate the epithelial fragments for 1 h at RT in blocking solution.

***Note:*** This can also be done for 8–12 h (overnight) at 4°C.

13. Carefully remove blocking solution and incubate the epithelial fragments with primary antibody diluted in blocking solution for 8–12 h (overnight) or over several days at 4°C. Use Rat anti-CD49f (1:500) for basal and Rabbit anti-KLF4 (1:400) for suprabasal cells. Troubleshooting 3.
14. Wash the tissue samples at 20°C–23°C for at least 2 h in washing solution (0.2% Tween-20 in 1 x DPBS) changing the washing buffer every 30 min. Place the plate on a rocker shaker to improve washing efficiency.
15. Prepare the EdU detection reaction mix 10 min before the last washing step is finished. Use a new 24 well-plate and pipette 300 μl of EdU reaction mix to as many wells as there are tissue pieces.

**CRITICAL:** Some antibodies need to be added AFTER the EdU development (i.e. anti-GFP). Carefully read about the compatibility of the antibody with EdU detection. Keep in the dark. Troubleshooting 4.

16. Carefully transfer the esophageal tissue fragments from the washing solution to the EdU reaction mix.
17. Incubate at 20°C–23°C for 30 min.

***Note:*** From now on incubate in a dark room or cover the plate with aluminum foil to protect the fluorophore from light exposure.

18. Wash the tissue samples at 20°C–23°C for at least 1 h in washing solution changing the solution every 30 min. Place the plate on a rocker shaker while washing.
19. Incubate the samples with secondary antibody and 1.25 μg/mL DAPI (diluted in blocking solution) for 2 h at 20°C–23°C or for 8–12 h (overnight) at 4°C. Secondary antibodies are commonly Donkey anti Rabbit (use 1:1000) or Donkey anti Rat (use 1:1000). Troubleshooting 5.
20. Wash samples at 20°C–23°C in washing solution for at least 2 h, changing solution carefully every 30 min. Place the plate on a rocker shaker while washing.
21. Place the tissues on the slide with the basal layer side facing upwards.

***Note:*** The incision or cut made with a scalpel (step 8) helps to orient the tissue with the basal side facing up.

22. Mount the tissue with ProlongGold antifade. Depending on the size of your tissue piece use 15–25 μl of mounting medium.

***Note:*** ProlongGold is a hard-setting liquid media that does not significantly quench the initial fluorescence signal to protect fluorescent dyes from fading (photobleaching) during fluorescence microscopy experiments which additionally allows for longer term storage of samples.

23. Carefully add a coverslip and let the slide rest in the dark at 20°C–23°C for around 1 h to let the medium harden. Troubleshooting 6. Mounted slides can be stored at 4°C for up to two years retaining fluorescence.

***Note:*** No spacer is needed to preserve the 3-D structure since the esophageal epidermis is thin. However, ensure not to apply too much pressure on the coverslip when mounting. 15–30 μl mounting medium is sufficient to support the coverslip without crushing the tissue and simultaneously prevents the tissue from floating when applying the coverslip.

24. Go to an inverted confocal and image!

## Expected outcomes

**Figure 3:**
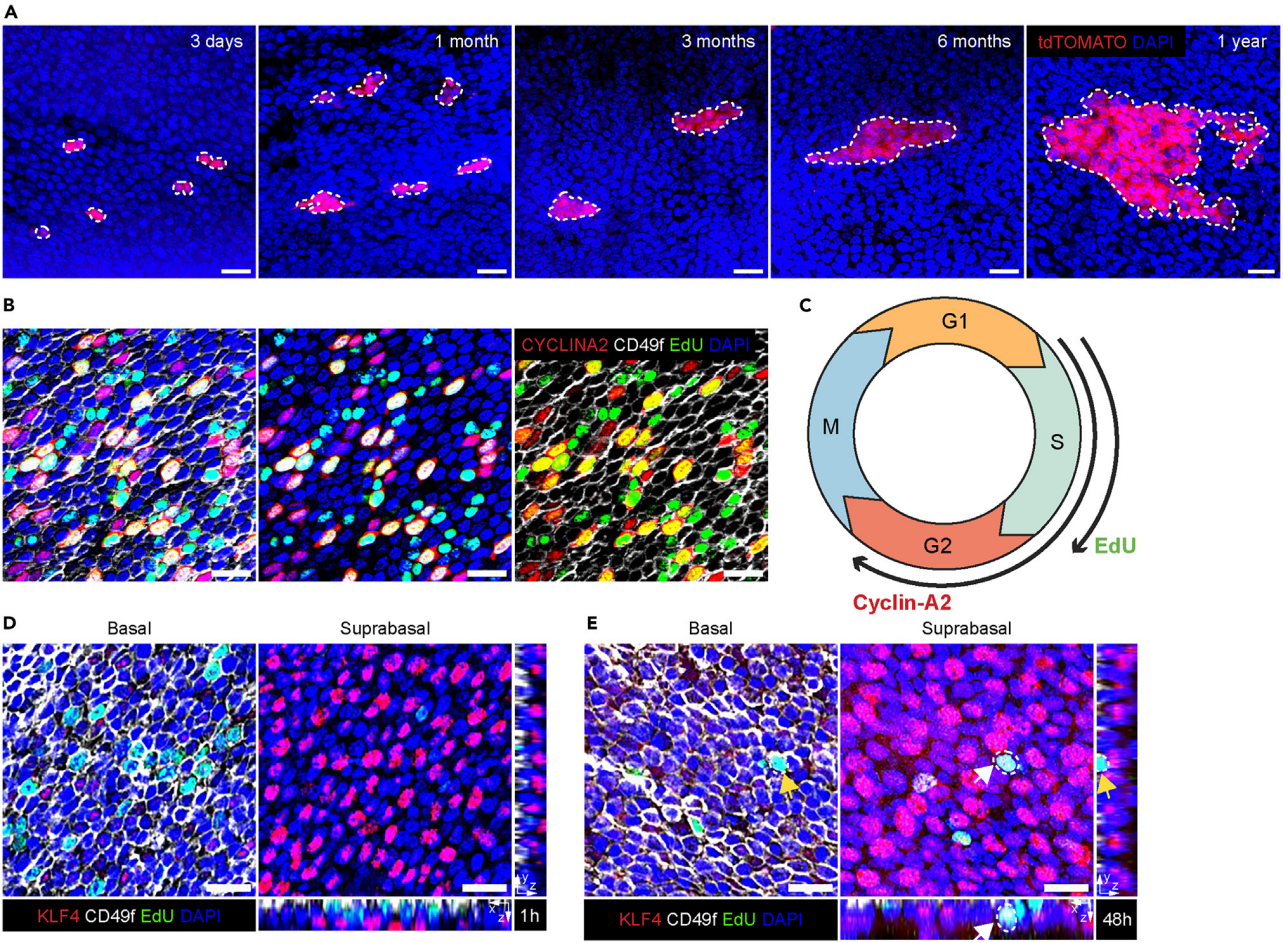
Representative images of lineage tracing and immunofluorescent labeling (A) Timeline displaying representative images of recombined Troy^CreERT2^;tdTomato-positive, basal clones up to one year after tamoxifen induction. Dotted lines indicate clonal border. Scale bars = 20 μm (B) Whole-mount images of CYCLINA2, CD49f and EdU allowing for the detection of specific cell-cycle phases in the basal layer progenitor cells. Scale bar = 20 μm. (C) Illustration of the cell-cycle displaying incorporation of EdU into DNA during S-phase and CYCLINA2 expression during S- and G2-phase. (D) Whole-mount images including an orthogonal view of the esophageal epithelium after a 1-h EdU pulse. Basal cells are labeled with CD49f and suprabasal cells with KLF4. EdU-positive cells are located in the basal layer. Scale bar = 20 μm. (E) Whole-mount images including an orthogonal view of the esophageal epithelium 48-h after EdU administration. EdU-positive cells are found in the suprabasal layer, co-expressing KLF4. Scale bar = 20 μm.

In contrast to cross sections, whole mounts allow users to greatly expand on the number of basal and suprabasal cells possible to capture in one image. Preparing whole mounts from the entire esophageal axis is also feasible, rendering detection and quantification of scarce epithelial clones or cell populations possible. Herein, we provide examples of lineage tracing data from whole mounts, visualizing basal clone expansion with time (Figure 3A). Studies from the esophageal epithelium demonstrate remaining clones after more than one year of lineage tracing,^1^^,^^2^^,^^3^ where tissue contribution of recombined cells normally remain constant with time – a hallmark of neutral competition. However, inducing oncogenic mutations is known to skew clone behavior.^4^^,^^8^ In Grommisch et al.,^1^ we investigated short term clonal data upon all-trans retinoic acid treatment and found a differential response within distinct progenitor subpopulations. Lineage tracing followed by subsequent clone and cell distribution analysis in epithelial whole mounts represents a powerful method for understanding *in vivo* cell behaviors, normally and during tissue stress.

In Figure 3B we display successful EdU incorporation and CYCLINA2 protein detection in basal cells. Combining EdU with additional cell cycle stage specific markers enables detection of specific cell cycle stages. Here we demonstrate the separation of G2 and S-phase progenitor cells. Furthermore, we demonstrate how EdU can be used to determine stratification states (Figures 3D and 3E). A short 1-h pulse of EdU can be used to determine the proliferation rate of esophageal progenitor cells after different treatments. We show that 1 h after EdU administration, all EdU-positive cells reside in the basal layer. In contrast, when introducing a 48-h chase period, EdU-positive cells are co-labeled with suprabasal differentiation markers such as KLF4, indicating that they have committed to differentiation. Combining this type of EdU-tracing with lineage tracing in reporter mouse lines or after tissue stress will be powerful in understanding epithelial differentiation dynamics.

## Limitations

Certain antibodies will require longer fixation times for appropriate detection while other antibodies work best on shortly fixed samples. Therefore, not all antibodies can be counterstained simultaneously. Make sure to titer the amount of antibody necessary and optimize the staining conditions regarding fixation and incubation time for each antibody, respectively. If using this protocol in settings where tissue homeostasis is perturbed, thickening of the esophageal epithelium or wound response may impact antibody penetration.

## Troubleshooting

### Problem 1

The epithelium and submucosa are not separable after EDTA incubation (Related to steps 6–8).

### Potential solution

Ensure that enough liquid is covering the tissue samples and place the plate on a rocker shaker at 37°C. Double check that the esophageal pieces do not get stuck to the walls of the wells of the plate during incubation. Extend the incubation time. Sometimes 2 h incubation is not sufficient. Try the separation on a single esophageal fragment first and increase the incubation time accordingly if the separation was not successful.

### Problem 2

The epithelium ruptures following EDTA incubation (Related to steps 6–8).

### Potential solution

Shorten the incubation time with EDTA. Try to separate the epithelium and submucosa already after 2–2.5 h of EDTA incubation. Too long incubations can render the tissue handling more difficult and should be avoided. Extending incubation with EDTA beyond 3 h is not recommended.

### Problem 3

The staining intensity of the esophageal samples is not uniform and stronger at the edge of the tissue segments. This is relevant for both primary antibodies and EdU (Related to steps 16–18).

### Potential solution

• A prolonged incubation time of the primary antibody yields clearer and more uniform signal. This is especially evident when using antibodies directed against transcription factors such as KLF4. Ensure that enough liquid is added to cover the tissue pieces and place the well plate on a rocker shaker at 4°C. Primary antibody incubations for up to 72 h will lead to improved results regarding intensity and uniformity of the staining. In addition, titer the antibody of choice when using for the first time. Information regarding recommended concentrations can normally be found on the manufacturer’s website or product information sheet.
• Instead of the homemade EdU detection reaction it is possible to use Invitrogen Click-iT EdU Cell Proliferation Kit for Imaging. Follow the manufacturer’s protocol for best result.

### Problem 4

EdU signal intensity is low or undetectable (Related to steps 15–17).

### Potential solution

Always inject several animals to exclude the possibility of a failed i.p. injection. Longer fixation times (up to 8–12 h (overnight) fixation)) normally help to increase EdU signal intensity, but increasing fixation time may compromise the labeling of other antibodies.

When developing the EdU signal using the Click-iT reaction, always make sure that enough liquid is covering the tissue fragments, that the EdU reaction buffer is prepared with fresh components and not more than 15 min before usage. After the Click-iT reaction is finished, keep samples in the dark during incubation times (wrap plates in aluminum foil or keep in closed containers) and avoid bright light during tissue handling to prevent bleaching of the EdU signal.

### Problem 5

The DAPI intensity or staining intensity of the esophageal samples is not uniform and stronger at the edge of the epithelial segments (Related to step 19).

### Potential solution

Combine the 8–12 h (overnight) secondary antibody incubation at 4°C (or 3-h incubation at 20°C–23°C) with the DAPI counterstain. Ensure that the liquid is covering the tissue piece and place the plate on a rocker shaker.

### Problem 6

The epithelial cell fragments are compressed upon mounting (Related to steps 21–23).

### Potential solution

It is crucial to not apply too much force when mounting the tissue fragments to avoid compression and subsequent tissue disruption and cell displacement. Make sure to use enough mounting medium and try not to apply pressure when adding the coverslip. The use of spacers can aid the mounting of a tissue piece to a confined area and depth. However, the esophageal epithelium should be thin enough to successfully mount samples without the use of spacers.

## Resource availability

### Lead contact

Further information and requests for resources and reagents should be directed to and will be fulfilled by the lead contact, Maria Genander (maria.genander@ki.se).

### Technical contact

Technical questions on executing this protocol should be directed to and will be answered by the technical contact, David Grommisch (david.grommisch@mpi-cbg.de).

### Materials availability

No new materials were generated in this study.

### Data and code availability

Grommisch et al.^1^ includes all datasets generated or analyzed during this study.

## Acknowledgments

The establishment of esophageal epithelial whole mounts was first reported by Philip H Jones’ lab.^2^ This study was supported by ERC Starting Grant (TroyCAN 851241), Cancerfonden (19 0007Pj), SFO StratRegen 2023/24 Junior Grant, and Vetenskapsrådet (2023-02743). M.G. is a Cancerfonden Senior Investigator. We are grateful for the support of the Biomedicum Imaging Core for technical assistance. We thank all members of the Genander lab for constructive feedback on the manuscript.

## Author contributions

D.G. and M.G. conceived the project, designed the experiments, and wrote the manuscript. D.G. and W.Y. performed the experiments.

## Declaration of interests

The authors declare no competing interests.
